# Efficacy of intraoperative irrigation with artificial cerebrospinal fluid in chronic subdural hematoma surgery: study protocol for a multicenter randomized controlled trial

**DOI:** 10.1186/s13063-023-07889-7

**Published:** 2024-01-02

**Authors:** Yoshitaka Nagashima, Yoshio Araki, Kazuki Nishida, Shunichiro Kuramitsu, Kenichi Wakabayashi, Shinji Shimato, Takeshi Kinkori, Toshihisa Nishizawa, Takahisa Kano, Toshinori Hasegawa, Atsushi Noda, Kenko Maeda, Yu Yamamoto, Osamu Suzuki, Naoki Koketsu, Takeshi Okada, Masashige Iwasaki, Kiyo Nakabayashi, Shigeru Fujitani, Hideki Maki, Yachiyo Kuwatsuka, Masahiro Nishihori, Takafumi Tanei, Tomohide Nishikawa, Yusuke Nishimura, Ryuta Saito, Tokai Neurosurgical disorder Research Group CSDH Study investigators, Satoshi Maesawa, Takashi Izumi, Kazuya Motomura, Eiji Ito, Eriko Okumura, Fumiharu Ohka, Kazuhito Takeuchi, Yuichi Nagata, Kinya Yokoyama, Tomotaka Ishizaki, Fumiaki Kanamori, Yutaro Fuse, Aya Sato, Seki Yukio, Tetsuya Nagatani, Yusuke Sakamoto, Kenji Uda, Tetsuya Tsukada, Takayuki Ishikawa, Hiroo Sasaki, Ienaga Jyunpei, Yosuke Sakai, Toru Watanabe, Yuichiro Isozawa, Nobuyasu Kato, Yasukazu Kajita, Noriyuki Suzaki, Kaoru Eguchi, Masashi Ito, Shunsaku Goto, Ryo Ando, Hayato Yokoyama, Kazuichi Terao, Naoto Kawaguchi, Yu Sugiyama, Hitofumi Oyama, Tomomi Kawaguchi, Takafumi Otsuka, Taiko Osada, Tomoki Matsuyama, Kengo Hirayama, Kouki Takeda, Shohei Mizuno, Kazuhiko Watanabe, Shigekazu Nakamura, Shinji Ota, Naoki Kato, Masahiro Ueno, Yusuke Sato, Masato Otawa, Mizuki Nakano, Yuhei Takido, Wataru Okada, Takashi Sakuma, Shun Yamamoto, Chihiro Iinuma, Takumi Asai, Yoshihiro Yamashita, Shinsuke Muraoka, Shigeaki Nawa, Hajime Hamasaki, Takahiko Fukui, Naoki Suzuki, Ikuo Takahashi, Ota Keisuke, Hirotada Kataoka, Yosuke Tamari, Tomoya Goto, Toshiki Seki, Tomiyuki Miyake, Takenori Kato, Takehiro Naito, Akihiro Mizuno, Yuta Koketsu, Takumi Otake, Akinori Kageyama, Hirotaka Niwa, Hiroyuki Oishi, Toshio Yokoe, Shuntaro Takasu, Masaya Takemoto, Jungsu Choo, Mizuka Ikezawa, Fumihiro Sago, Daiki Somiya, Mizuki Terano, Kohei Doba, Daimon Shiraishi, Sho Akahori, Motonori Ishii, Nobuhisa Fukaya, Toshiki Fukuoka, Takayuki Awaya, Shoko Wakisaka, Masao Tambara, Hiroyuki Shimizu, Satoshi Shinoda, Yusuke Muramatsu, Otone Endo, Kentaro Fujii, Yiichi Kawasaki, Takato Ono, Kento Hirayama, Yuya Itagaki, Shuya Kurono, Jyunzo Ishiyama, Chihiro Aoshima, Yuki Sunohara, Yuri Aimi, Mitsuhiro Yoshida, Mamoru Ishida, Masayuki Kimata, Hisashi Mizutani, Kenichi Hattori, Kentaro Wada, Takashi Mamiya, Masahiro Wakabayashi, Toshiaki Hirose, Risa Ito, Yusuke Ota, Hiroaki Kubo, Tomoyuki Noda, Teppei Kawabata, Tasuku Imai, Takahiro Oyama, Kei Hachiya, Yasumasa Nishida

**Affiliations:** 1 Department of Neurosurgery, Nagoya University Graduate School of Medicine, Nagoya, Japan; 2 Department of Neurosurgery, Japanese Red Cross Aichi Medical Center Nagoya Daini Hospital, Nagoya, Japan; 3 Department of Advanced Medicine, Nagoya University Hospital, Nagoya, Japan; 4 Department of Neurosurgery, National Hospital Organization Nagoya Medical Center, Nagoya, Japan; 5 Department of Neurosurgery, Toyohashi Municipal Hospital, Toyohashi, Japan; 6 Department of Neurosurgery, Handa City Hospital, Handa, Japan; 7 Department of Neurosurgery, Okazaki City Hospital, Okazaki, Japan; 8 Department of Neurosurgery, Kariya Toyota General Hospital, Kariya, Japan; 9 Department of Neurosurgery, Anjo Kosei Hospital, Anjo, Japan; 10 Department of Neurosurgery, Komaki City Hospital, Komaki, Japan; 11 Department of Neurosurgery, Nishio Municipal Hospital, Nishio, Japan; 12 Department of Neurosurgery, JCHO Chukyo Hospital, Nagoya, Japan; 13 Department of Neurosurgery, Inazawa Municipal Hospital, Inazawa, Japan; 14 Department of Neurosurgery, Nagoya Ekisaikai Hospital, Nagoya, Japan; 15 Department of Neurosurgery, Tosei General Hospital, Seto, Japan; 16 Department of Neurosurgery, Kainan Hospital, Yatomi, Japan; 17 Department of Neurosurgery, Shizuoka Saiseikai General Hospital, Shizuoka, Japan; 18 Department of Neurosurgery, Yokkaichi Municipal Hospital, Yokkaichi, Japan; 19 Department of Neurosurgery, Japanese Red Cross Aichi Medical Center Nagoya Daiichi Hospital, Nagoya, Japan; 20 Department of Neurosurgery, Ogaki Municipal Hospital, Ogaki, Japan

**Keywords:** Artificial cerebrospinal fluid, Chronic subdural hematoma, Irrigation fluid, Recurrence, Surgical evacuation

## Abstract

**Background:**

The surgical techniques for treatment of chronic subdural hematoma (CSDH), a common neurosurgical condition, have been discussed in a lot of clinical literature. However, the recurrence proportion after CSDH surgery remains high, ranging from 10 to 20%. The standard surgical procedure for CSDH involves a craniostomy to evacuate the hematoma, but irrigating the hematoma cavity during the procedure is debatable. The authors hypothesized that the choice of irrigation fluid might be a key factor affecting the outcomes of surgery. This multicenter randomized controlled trial aims to investigate whether intraoperative irrigation using artificial cerebrospinal fluid (ACF) followed by the placement of a subdural drain would yield superior results compared to the placement of a subdural drain alone for CSDH.

**Methods:**

The study will be conducted across 19 neurosurgical departments in Japan. The 1186 eligible patients will be randomly allocated to two groups: irrigation using ACF or not. In either group, a subdural drain is to be placed for at least 12 h postoperatively. Similar to what was done in previous studies, we set the proportion of patients that meet the criteria for ipsilateral reoperation at 7% in the irrigation group and 12% in the non-irrigation group. The primary endpoint is the proportion of patients who meet the criteria for ipsilateral reoperation within 6 months of surgery (clinical worsening of symptoms and increased hematoma on imaging compared with the postoperative state). The secondary endpoints are the proportion of reoperations within 6 months, the proportion being stratified by preoperative hematoma architecture by computed tomography (CT) scan, neurological symptoms, patient condition, mortality at 6 months, complications associated with surgery, length of hospital stay from surgery to discharge, and time of the surgical procedure.

**Discussion:**

We present the study protocol for a multicenter randomized controlled trial to investigate our hypothesis that intraoperative irrigation with ACF reduces the recurrence proportion after the removal of chronic subdural hematomas compared with no irrigation.

**Trial registration:**

ClinicalTrials.gov jRCT1041220124. Registered on January 13, 2023.

**Supplementary Information:**

The online version contains supplementary material available at 10.1186/s13063-023-07889-7.

## Introduction

### Background and rationale {6a}

Chronic subdural hematoma (CSDH) is a neurological condition caused by a hematoma that slowly collects in the subdural space between the dura mater and brain. CSDH is one of the most common neurosurgical disorders, particularly in the elderly population [1, 2]. As the world population ages, the incidence and median age of patients with CSDH steadily increase [3, 4]. CSDH presents with a variety of neurological symptoms, ranging from mild symptoms such as headache and dizziness to severe symptoms like disorientation. In cases with minimal or no symptoms, the hematoma may resolve spontaneously, while in severe cases, surgery is indicated when the symptoms progress or the volume of the hematoma is large [5, 6]. Surgical outcomes are generally favorable, but hematoma regrows in 10–20% of cases postoperatively and necessitates reoperation [1]. Therefore, appropriate surgical procedures must be established to reduce postoperative recurrence.

The focus of this study stems from the debate over the efficacy of intraoperative irrigation — a technique involving washing out the hematoma during surgery — in preventing the hematoma recurrence. Some studies suggest that irrigation significantly reduces recurrence rates, while others report no difference or even that perfusion should be avoided [7–16]. A meta-analysis including some of these studies revealed no significant difference in recurrence or complications between the two methods [17]. As a reason for these inconclusive results, variability in the type of irrigation fluid used can be considered. Specifically, in previous studies, artificial cerebrospinal fluid (ACF) (Table 1) was not used when washing out hematomas. Recent reports suggest that ACF is more effective in reducing recurrence rates compared to normal saline [18]. Therefore, a more definitive investigation is needed regarding the role of intraoperative irrigation in the management of CSDH when appropriate perfusion fluids are used.

**Table 1:** Composition of human spinal fluid and solutions used in neurosurgery

Component	Normal human CSF	ACF	NS	LR
Na^+^ (mEq/L)	145	145	154	130
K^+^ (mEq/L)	2.8	2.8	0	4
Mg^2+^ (mEq/L)	2.2	2.2	0	0
Ca^2+^ (mEq/L)	2.3	2.3	0	3
Cl^−^ (mEq/L)	111.9	129	154	109
P (mmol/L)	1.1	1.1	0	0
HCO^3−^ (mEq/L)	23.1	23.1	0	0
Lactate^−^ (mEq/L)	0	0	0	28
Glucose (g/L)	0.61	0.61	0	0
Osmolality ratio	≈1	≈1	1	≈0.9
pH	7.307	≈7.3	≈6.3	≈6.7

*Abbreviations*: *CSF* cerebrospinal fluid, *ACF* artificial cerebrospinal fluid, *NS* normal saline, *LR* lactated Ringer’s solution

Furthermore, various factors like age, antithrombotic therapy, alcoholism, and pre- and postoperative computed tomography (CT) have been identified as recurrence risks, underscoring the need for a comprehensive approach to studying CSDH recurrence [19–25].

### Objectives {7}

We hypothesized that with the use of appropriate irrigation fluid, CSDH surgery with irrigation would reduce the recurrence rate compared to the non-irrigation group. The primary objective of this study is to determine whether intraoperative irrigation with ACF or no irrigation is associated with better clinical outcomes and recurrence proportions of CSDH. This will be measured primarily by the rate of meeting criteria for symptomatic CSDH recurrence necessitating reoperation within a 6-month period post-surgery. Furthermore, this study seeks to explore the potential of irrigation as a more effective intervention for patients at high risk of CSDH recurrence, thereby providing a more holistic understanding of the condition's management by stratifying the CSDH patient background.

### Trial design {8}

This study is a prospective, multicenter randomized, controlled, non-blinded trial. This study evaluates intraoperative irrigation via ACF with a subdural drain versus a subdural drain alone after the evacuation of a CSDH. Except for randomization to irrigation versus no irrigation, the management of the study participants will not differ from the current management of patients. A 1:1 stratified randomization will be performed at each site using a web-based system. Clinical symptoms and imaging assessments will be conducted by the treating physicians at each facility, following the timeline of the participants.

## Methods: participants, interventions and outcomes

### Study setting {9}

The study will recruit patients with CSDHs requiring surgery at hospitals in the Tokai region of Japan. The participating sites are the neurosurgical departments of the National Hospital Organization Nagoya Medical Center (Nagoya, Japan), Kariya Toyota General Hospital (Kariya, Japan), Anjo Kosei Hospital (Anjo, Japan), Komaki City Hospital (Komaki, Japan), Toyohashi Municipal Hospital (Toyohashi, Japan), Okazaki City Hospital (Okazaki, Japan), Nishio Municipal Hospital (Nishio, Japan), Inazawa City Hospital (Inazawa, Japan), Handa City Hospital (Handa, Japan), Nagoya Ekisaikai Hospital (Nagoya, Japan), Tosei General Hospital (Seto, Japan), Shizuoka Saiseikai General Hospital (Shizuoka, Japan), Japan Community Health Care Organization Chukyo Hospital (Nagoya, Japan), Yokkaichi Municipal Hospital (Yokkaichi, Japan), Kainan Hospital (Yatomi, Japan), Japanese Red Cross Aichi Medical Center Nagoya Daiichi Hospital (Nagoya, Japan) Japanese Red Cross Aichi Medical Center Nagoya Daini Hospital (Nagoya, Japan), Ogaki Municipal Hospital (Ogaki, Japan), and Nagoya University Hospital (Nagoya, Japan).

### Eligibility criteria {10}

#### Study participants

Patients diagnosed with CSDH for whom surgical resection is indicated will be screened for participation.

#### Inclusion criteria

- Patients with symptomatic CSDH requiring burr-hole evacuation
- Patients > 20 years
- Patients undergoing surgery for single-sided CSDH

#### Exclusion criteria

- Patients who have previously undergone a craniotomy
- Patients who have previously received an ipsilateral craniostomy
- Patients with spinal fluid shunting
- Patients with intracranial mass lesions that may affect their current symptoms (e.g., very small tumors or contralateral hematomas) should be excluded.
- Patients who have received radiation or chemotherapy within the last 5 years
- Immunocompromised states
- Patients with such severe thrombotic risk from the discontinuation of antithrombotic drugs are not tolerated (recent cardiac or intracranial stents, recent pulmonary embolisms, and mechanical valves).
- Patients deemed unable to insert a drain preoperatively due to a lack of patience or small hematoma
- Patients who, in the judgment of the investigator, are likely to be non-compliant or uncooperative during the study.

### Who will take informed consent? {26a}

In this study, a neurosurgeon, who is also an investigator or subinvestigator, approaches patients who are eligible for CSDH surgery. Following the explanation and consent for surgery, patients are informed about the study. Patients are enrolled in the study only if they consent to both the surgery and the research. If a patient is unable to provide written consent, it will be obtained from their next of kin. Patients who choose not to participate in the study can still receive the same quality treatment as participants. The participants will be free to withdraw from the study at any time in accordance with the most recent 2013 version of the Declaration of Helsinki.

### Additional consent provisions for collection and use of participant data and biological specimens {26b}

We do not currently plan to use samples or information obtained from participants in this research for future studies. If this changes, we will obtain proper ethical approval, document the details clearly, and either secure consent from the participants or provide an opportunity to opt out by making the research information public before using the information in new research.

## Interventions

### Explanation for the choice of comparators {6b}

To evaluate the effectiveness of irrigation using an ACF following the removal of a CSDH, the intervention is to be divided into two groups: one that will undergo hematoma irrigation and another that will not. The most widely and commercially available ACF for neurosurgical procedures in Japan (ARTCEREB Cerebrospinal Surgery Perfusion and Irrigation Solution®, Otsuka Pharmaceutical Factory, Tokushima, Japan) is chosen as the irrigation fluid in this study. Except for washing out the hematoma intraoperatively, all perioperative management will be performed identically across both groups.

### Intervention description {11a}

In the hematoma irrigation group, the subdural space will be irrigated repeatedly until fluid drainage becomes clear, using at least 200 mL of ACF after CSDH removal. A closed drain will then be inserted and left in the cavity for at least 12-h post-surgery.

In contrast, in the non-irrigation group, a drain will be placed without irrigation after performing the burr-hole craniostomy. Similar to the hematoma irrigation group, a closed drain will be left in the cavity for at least 12-h post-surgery.

Previous literature has examined the direction and duration of subdural space drainage, yet a consensus on evidence-based guidelines remains elusive [26–28]. Therefore, this study advocates that the minimum duration of subdural drainage should be at least 12 h, and that the exact position and duration of the drain should be left to the discretion of the attending physician. Similarly, as the optimal volume for irrigation is not yet known [29], the practical volume used in this study is also left to the discretion of the attending physician, with a minimum volume of 200 ml. This approach highlights the importance of clinical judgment in the absence of conclusive evidence and ensures flexibility in patient care while adhering to minimum standards.

### Criteria for discontinuing or modifying allocated interventions {11b}

After randomization, patients who wish to change their assigned surgical procedure can request an intervention change and withdraw from the trial. Patients do not have to provide the reason for withdrawal. If the surgeon intentionally or unintentionally administers a treatment different from the assigned group after randomization, the case will be excluded from the analysis, and crossover of participants is not permitted. This approach ensures the integrity of the trial and respects patient autonomy.

### Strategies to improve adherence to interventions {11c}

This study required the investigators to provide data on whether they performed the treatment assigned to them. Information on the type, quantity, and temperature of the solutions used for irrigation is to be collected.

### Relevant concomitant care permitted or prohibited during the trial {11d}

Reoperation at a stage that does not meet the criteria for reoperation is prohibited. All other medical treatments are permitted.

### Provisions for posttrial care {30}

This study will implement and collect data on treatments accepted by national insurance in Japan. Therefore, it is not compensable to those likely to suffer harm from trial participation, as per clinical research insurance, and is handled the same as normal medical treatment.

### Outcomes {12}

#### Assessment items

The demographic information collected includes age, sex, medical history, lifestyle history, and traumatic episodes that may have contributed to the condition. Patients’ living situations, whether at home, in a hospital, rehabilitation center, or nursing home, are to be recorded as well.

Neurological status is to be assessed using the modified Rankin Scale (mRS), Glasgow Coma Scale (GCS), Markwalder classification, and any existing neurological symptoms. The modified Rankin Scale (mRS) was developed to evaluate the severity of stroke outcomes. However, its application extends to clinical assessments of CSDH [30–32]. This scale ranges from 0, denoting the absence of symptoms, to 6, which signifies death (Table 2). GCS is used to objectively express the degree of impairment of all types of consciousness in terms of the three aspects of eye-opening, motor, and verbal reactions (Table 3). The Markwalder classification divides the severity of symptoms of CSDH into 0–4 [33] (Table 4). The scores on these scale at will be determined by physicians who were trained to obtain them.

**Table 2:** Modified Rankin Scale (mRS)

Score	Description
0	No symptoms
1	No significant disability: Despite symptoms, able to carry out all usual duties and activities
2	Slight disability: Unable to perform all previous activities but able to look after own affairs without assistance
3	Moderate disability: Requiring some help but able to walk without assistance
4	Moderately severe disability: Unable to walk without assistance and unable to attend to own bodily needs without assistance
5	Severe disability: Bedridden, incontinent, and requiring constant nursing care and attention
6	Death

**Table 3:** Glasgow Coma Scale (GCS)

Eye opening		Verbal response		Motor response	
Score	Description	Score	Description	Score	Description
4	Eyes open spontaneously	5	Orientated	6	Obeys commands
3	Eye opening to sound	4	Confused	5	Localizing pain
2	Eye opening to pain	3	Inappropriate words	4	Withdrawal from pain
1	No eye opening	2	Incomprehensible sounds	3	Abnormal flexion to pain
		1	No verbal response	2	Abnormal extension to pain
				1	No motor response

**Table 4:** Markwalder classification

Grade	Description
0	Patient neurologically normal
1	Patient alert and oriented; mild symptoms such as headache; absent or mild neurological deficit, such as reflex asymmetry
2	Patient drowsy or disoriented with variable neurological deficit, such as hemiparesis
3	Patient stuporous but responding appropriately to noxious stimuli; severe focal signs, such as hemiplegia
4	Patient comatose with absent motor responses to painful stimuli; decerebrate or decorticate posturing

Based on cranial CT imaging, the architecture of the CSDH, volume of the hematoma, and midline shift will be assessed. The hematoma architecture is classified into four distinct types according to the framework established by Nagaguchi et al.: homogeneous, laminar, separated, and trabecular [21]. The homogeneous type is characterized by a uniform density that varies from low to high. In contrast, the laminar type, a variation of the homogeneous, is distinguished by a thin, high-density layer along the inner membrane. The separated type comprises two distinct density zones with a clear demarcation, typically a lower density component located above a higher density component. The ‘gradation’ subtype with ambiguous boundaries is also included in the separated type. Finally, the ‘trabecular’ type presents inhomogeneous contents and a high-density septum running between the inner and outer membrane on a low-density to isodense background. The hematoma volume was calculated from the maximum width (A), length (B), and height (C) using the formula A × B × C/2 [34]. The midline shift is measured as the deviation of the midline at the slice where the foramen of Monroe can be seen on the CT axial image.

Medications are divided into those that may impact the recurrence of CSDH, such as antithrombotic agents, steroids, statins, and goreisan, and those that do not. Surgical details included surgeon experience, surgery duration, irrigation fluid volume, direction of the inserted drain, and temperature of the irrigation fluid (room temperature or equivalent to body temperature).

Adverse events are to be documented when they occur. Personally identifiable information, such as name and address, will not be recorded on REDCap® data to ensure the anonymity of individual participants.

### Primary endpoint

The primary endpoint is the proportion of patients who meet the criteria for ipsilateral reoperation within 6 months of surgery.

### Criteria for reoperation

Reoperation is indicated when the following two criteria are simultaneously fulfilled:

(i) Neurological symptoms are present that are considered to be caused by ipsilateral CSDH.
(ii) The hematoma has increased in size compared to immediately after surgery and compressed the brain parenchyma in CT scans.

### Secondary endpoints

- The proportion of reoperations within 6 months
- The proportion of reoperation stratified by preoperative hematoma architecture determined by CT scans
- Modified Rankin Scale, Glasgow Coma Scale, and Markwalder classification at 6 months
- Mortality at 6 months
- Complications related to the operation
- Length of hospital stay from surgery to discharge (including time spent in a rehabilitation unit)
- Duration of the surgical procedure

### Participant timeline {13}

**Fig. 1:**
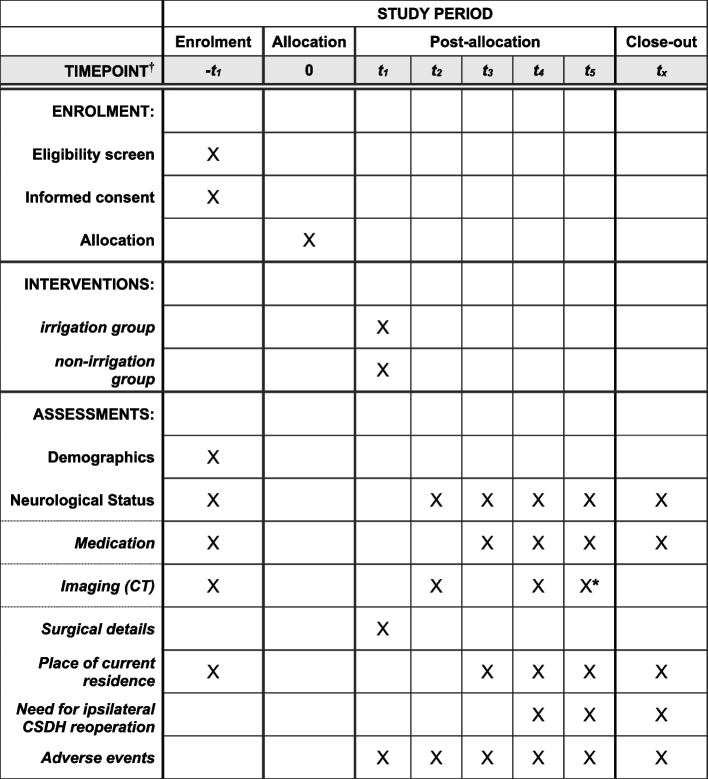
Checklist table. †Timepoint =  − *t*_1_, baseline (before surgery); 0, allocation; *t*_1_, surgery; *t*_2_, within 72 h after surgery; *t*_3_, at admission; *t*_4_, 4–6 weeks after surgery; *t*_5_, 6 months after surgery. *If reoperation has not been performed at 6 months postoperatively, imaging studies must be performed in patients with worsening neurological symptoms. Radiological imaging is not always necessary if the patient’s symptoms are stable

Data will be recorded in Research Electronic Data Capture (REDCap®) preoperatively, intraoperatively, within 72 h (postoperatively), at hospital admission, 4–6 weeks (postoperatively), and 6 months (postoperatively) (Fig. 1).

### Sample size {14}

The present study aimed to recruit 1186 cases, with 593 patients allocated to each group. The target number of cases was determined based on a previous meta-analysis that examined the efficacy of irrigation for CSDH. A meta-analysis reports a recurrence proportion of 8.06% (38/471) in the irrigation group and 13.05% (59/452) in the non-irrigation group; however, this difference was not statistically significant [17]. The 95% confidence intervals were 5.8–10.9% for the irrigation group and 10.1–16.5% for the non-irrigation group. To account for the differences in outcome measures between the prior and present studies, we set the proportions of patients meeting the criteria for ipsilateral reoperation at 7% for the irrigation group and 12% for the non-irrigation group. Using *α* = 0.05 and *β* = 0.20 and accounting for a 10% dropout proportion, a total of 1186 cases, with 593 cases in each group, have been calculated to be necessary for the current study. In determining the sample size, the sample size calculation tool for 2 × 2 contingency tables by Kazuhiko Nagashima was used [35] (https://nshi.jp/contents/js/twofreq/).

### Recruitment {15}

All CSDH patients who meet the eligibility criteria are eligible to participate. If the doctors at each site cannot immediately determine whether a patient is eligible to participate, the Nagoya University research team will respond to queries.

## Assignment of interventions: allocation

### Sequence generation {16a}

Randomization was performed using a minimization method of balancing age (69 years or younger vs. 70 years or older), hematoma architecture on CT scans (homogeneous, stratified, isolated, and trabecular), the presence of antiplatelet therapy, preoperative GCS score (13 or lower vs. 14 or higher), and institution at baseline in a 1:1 ratio. If there is a small contralateral hematoma, only the hematoma architecture is considered when performing surgery.

### Concealment mechanism {16b}

Clinical information required for the minimization method would be inserted into a web-based registration system for allocation. This process will be performed before surgery, and no registrations will be accepted thereafter.

### Implementation {16c}

Registration and randomization of patients will be performed by the physician at each participating facility when the decision for surgery has been made and consent from the patient or proxy has been obtained. The physician at each site will perform the surgery according to the method allocated in the web system.

## Assignment of interventions: blinding

### Who will be blinded {17a}

Given the nature of surgical intervention, it is impossible to blind the medical staff involved. Furthermore, since the primary outcome measure is meeting the criteria for reoperation, blinding the participants would be meaningless. Therefore, from the perspective of protecting participants’ rights, participant blinding will not be implemented either.

### Procedure for unblinding if needed {17b}

Blinding will not be performed in this study.

## Data collection and management

### Plans for assessment and collection of outcomes {18a}

All data will be collected and entered online by investigators at each site using REDCap®. The physician at each site will document medical records based on the data collection items, and these records will be the primary source for input into REDCap®. The results will be verified by the Nagoya University research team during the study. If data has not been entered, an email will be sent to the investigators at each site to encourage them to enter the data.

### Plans to promote participant retention and complete follow-up {18b}

If the patient does not attend according to the established schedule, the study investigator will contact the patient or family member to ask about the situation and whether the investigator can provide assistance to avoid future missed sessions. If the patient cannot visit for the 6-month postoperative assessment of the primary outcome, the status would be checked telephonically.

### Data management {19}

The REDCap online database serves as a platform for gathering and storing data in an academic context, ensuring password-protected access for authorized researchers. Physicians at each site can only enter and review data for their own site. Only the Nagoya University research team has access to data from all sites. This database incorporates mandatory data entry fields to minimize missing data. It also offers features such as range checking of data values and question branching. The gathered data will be handled and stored in compliance with the management plan established according to Nagoya University’s Research Data Management Policy.

### Confidentiality {27}

The forms used to code the patients are stored at each site in a locked cabinet accessible only to the investigators and administrators responsible for the study. Individual patient-identifiable information will not be entered into the REDCap®.

### Plans for collection, laboratory evaluation, and storage of biological specimens for genetic or molecular analysis in this trial/future use {33}

No additional biological samples are collected from participants other than those taken as part of routine medical care.

## Statistical methods

### Statistical methods for primary and secondary outcomes {20a}

A statistician blinded to the treatment assignments will perform the analysis. For the primary endpoint, we will adhere to the intention-to-treat (ITT) principle; all patients who have been randomized and underwent surgery will be analyzed according to the group they were allocated to, irrespective of the treatment they received. When outcomes are missing for these patients, they will be treated as not having met the criteria for reoperation. The comparison for this endpoint between the two groups will be made using Fisher’s exact test. As a secondary analysis, the odds ratio estimates and their 95% confidence intervals will be calculated for the primary endpoint and secondary endpoints of the proportion of reoperations, the proportion of deaths, and the proportion of surgery-related complications. For the secondary endpoints of mRS, change in GCS, time from surgery to discharge, and operative time, the two groups will be compared by calculating the mean difference between the groups and its 95% confidence interval. 95% confidence intervals will be calculated using the Clopper-Pearson and bootstrap methods, respectively.

### Interim analyses {21b}

No interim analyses are planned.

### Methods for additional analyses (e.g. subgroup analyses) {20b}

Subgroup analyses or other exploratory analyses may be added if necessary.

### Methods in analysis to handle protocol nonadherence and any statistical methods to handle missing data {20c}

If a significant amount of data is missing for any of the secondary endpoints or other analyses, we will conduct exploratory analyses to assess the impact of the missing data.

### Plans to give access to the full protocol, participant level-data, and statistical code {31c}

The protocol for this clinical trial is publicly available. The data sets and statistical codes analyzed in this study are available from the corresponding author upon reasonable request. Prior to data lock, a statistical analysis plan (SAP) will be established. This plan is essential and will be promptly registered with the jRCT upon completion. All subsequent analyses will be conducted in accordance with this SAP.

## Oversight and monitoring

### Composition of the coordinating center and trial steering committee {5d}

The study is planned, conducted, and coordinated by the Nagoya University research team. Day-to-day support for the study will be provided by the Nagoya University research team’s coordination and management practitioner. The study’s conduct, safety, recruitment, and follow-up are to be reviewed monthly. The data management officer will manage the data appropriately and ensure quality, reliability, and integrity. Additionally, the Nagoya University research team will double-check the investigator’s decisions about whether patients meet the criteria for reoperation, the primary endpoint, before data locking.

### Composition of the data monitoring committee and its role and reporting structure {21a}

A monitor authorized by the Nagoya University School of Medicine will supervise the sources of consent forms and clinical data collected at each facility. Case registration will be temporarily suspended if the number of cases confirmed by monitoring shows discrepancies between the assigned treatment group and the irrigation information collected by REDCap® exceeds 5% of the target number of cases for this trial (59 cases). Subsequently, an independent monitoring committee consisting of three external experts who are not affiliated with this study will be consulted for advice on the feasibility of continuing this trial as well as on measures to prevent noncompliance with the assigned treatment group if the trial is to be continued. The independent monitoring committee has the following three members: Masahito Hara, Department of Neurosurgery, Aichi Medical University Graduate School of Medicine, Nagakute, Aichi, Japan; Norimoto Nakahara, Department of Neurosurgery, Nagoya Central Hospital; and Masasuke Ohno, Department of Neurosurgery, Aichi Cancer Center Hospital, Nagoya, Japan.

### Adverse event reporting and harms {22}

Expected adverse events include cerebral hemorrhage, ischemic stroke, wound infection, and meningitis, and all other adverse events will be collected. These adverse events will be rated in terms of severity on a 5-point scale from grades 1 to 5 based on the Common Terminology Criteria for Adverse Events (CTCAE). The investigator will immediately report any serious adverse events related to the clinical trial to the hospital director and the investigators managing the study (Y. N., R. S.). Data on all serious adverse events will also be collected in REDCap®. All data on adverse events will be published with the study results.

The principal investigator of the study will consider the continuation or discontinuation of the research under the following circumstances:

- If a serious adverse event or disease report suggests that continuing the study poses safety concerns
- If recruiting participants for the study becomes challenging, making it difficult to achieve the planned number of cases
- When significant new information regarding the quality, safety, or efficacy of the medication and surgical procedure is obtained
- If the certified clinical research review committee directs changes to the research plan and it is deemed difficult to comply with these changes

### Frequency and plans for auditing trial conduct {23}

There are no audits of study conduct planned for this study, other than monitoring.

### Plans for communicating important protocol amendments to relevant parties (e.g., trial participants, ethical committees) {25}

The research will be conducted following the most up-to-date version of the protocol. Any modifications made to the protocol document or informed consent form will be recognized as amendments. These amendments will be described and formally submitted for review and approval by the Nagoya University Ethics Committee.

## Dissemination plans {31a}

A comprehensive plan for publication and dissemination will be formulated, encompassing the delivery of research findings through conference presentations and the publication of peer-reviewed research articles.

## Discussion

This is to be the largest RCT investigating the effect of ACF irrigation on CSDH. Optimization of treatment strategies is required to reduce the recurrence proportion as the incidence of CSDH has been increasing worldwide, beyond Japan, owing to the aging population in recent years.

If the current study indeed confirms the recurrence proportion reduction with ACF irrigation, the surgical process for CSDH would be optimally achieved. This reduction in the need for reoperation would therefore significantly contribute to the maintenance of activities of daily living in geriatric care and ease the workload of medical staff.

The strength of this study lies in the large number of registered cases, with 1186 cases compared to previous trials, making it a considerably larger trial. The number of cases planned for enrollment is more than double that of an ongoing trial in Finland [36]. With such a large number of cases, the effectiveness of irrigation based on the risks of recurrence could be explored, including hematoma architecture on CT scans. CSDH is known to have varying proportions of recurrence based on preoperative clinical and imaging characteristics such as hematoma size, midline shift, and internal architecture [37]. Therefore, this study aims to investigate whether irrigation of hematomas with ACF is effective and is expected to provide a more personalized and effective approach for CSDH treatment. A limitation of this study is the possibility of bias due to the inability to blindly use irrigation fluid.

## Trial status

Patient enrollment would subsequently commence after each site’s permission to participate in the study was obtained. The first participant was enrolled in this study on January 31, 2023. The recruitment of participants is planned to be completed by June 30, 2025.

### Supplementary Information

**Additional file 1. **Model consent form.

## Data Availability

Only the designated investigators will have access to the final dataset.

## Abbreviations

ACF: Artificial cerebrospinal fluid
BTS: Butterfly needle tap and suction
CSDH: Chronic subdural hematoma
CT: Computed tomography
DPC: Diagnosis procedure combination
GCS: Glasgow Coma Scale
ICTRP: International Clinical Trials Registry Platform
RCT: Randomized controlled trial
ITT: Intention-to-treat
SAP: Statistical analysis plan
