# Supplementary material for: Efficacy of intraoperative irrigation with artificial cerebrospinal fluid in chronic subdural hematoma surgery: study protocol for a multicenter randomized controlled trial
Source: Trials. 2024 Jan 2;25:6. doi: 10.1186/s13063-023-07889-7 (PMC10759626; doi:10.1186/s13063-023-07889-7)
Supplement: Supplementary file 1 — Additional file 1. Model consent form. [file 13063_2023_7889_MOESM1_ESM.docx]

**Consent Form**

Principal Investigator: Professor Ryuta Saito, Department of Neurosurgery, Nagoya University School of Medicine

I have received an explanation using a document from [Name　　　　　　　　　　　　　　] regarding the research project "Study on the Impact of Hematoma Cavity Irrigation on Recurrence Rate during Chronic Subdural Hematoma Surgery" and have fully understood the following items (please check □ for the items you have been explained and understood):

□ The purpose, significance, implementation method, and anticipated risks of this research.

□ That not participating in this research will not disadvantage my future medical care.

□ That I can withdraw my consent at any time, even after agreeing initially.

□ That sufficient consideration will be given to the protection of personal information.

□ Methods of informing about analysis results, handling of intellectual property rights, and compensation for health damages.

I agree to participate in the research under the following conditions:

At the end of this research, regarding the samples provided:

About the information provided (please select one):

□ Please dispose of the information after the retention period ends.

□ I agree that the information provided may be used again for future clinical research planned and implemented, subject to new ethical review and approval by the head of the implementing institution.

Date: [Year　　] [Month　　　] [Day　　]

Name (Participant):

Name (Proxy):

Address:

（If this consent form is stored with documents that can identify the individual, it is not necessary to fill in the address.）

Relationship to the participant if a proxy:
